# QT prolongation and excessive variability predicts new-onset atrial fibrillation in the health screening data of Japanese adults

**DOI:** 10.1371/journal.pone.0333169

**Published:** 2025-10-22

**Authors:** Yuichi Ninomiya, Kei Enokizono, Shin Kawasoe, Takuro Kubozono, Hiroyuki Kamada, Yasuhisa Iriki, Yoshiyuki Ikeda, Hironori Miyahara, Koichi Tokushige, Mitsuru Ohishi

**Affiliations:** 1 Department of Cardiovascular Medicine and Hypertension, Kagoshima University Graduate School of Medical and Dental Sciences, Kagoshima, Japan; 2 Department of Cardiovascular Medicine, National Hospital Organization Kagoshima Medical Center, Kagoshima, Japan; 3 Kagoshima Kouseiren Hospital, Kagoshima, Japan; PearResearch / Government Doon Medical College, INDIA

## Abstract

**Background:**

A prolonged QT interval (QTc) is associated with the development of atrial fibrillation. However, reports linking QTc variability and atrial fibrillation are limited.

**Purpose:**

To investigate the relationship linking prolongation and variability of QTc with new-onset atrial fibrillation in Japanese adults.

**Methods:**

This retrospective study analyzed the annual health screening data of 103,304 adults (50,438 males; mean age, 54 years) who did not exhibit atrial fibrillation at baseline between April 2005 and October 2018. The majority of the study population underwent annual health examinations according to Japan’s health welfare policy. We calculated QTc times using the Bazett formula (QTc = QT/√RR). QTc variability is indicated by the gap between the maximum and minimum QTc values. Atrial fibrillation was diagnosed by 12-lead surface electrocardiography. The association between QT prolongation and variability in new-onset atrial fibrillation was ascertained by logistic regression analysis. The strength of the association was further analyzed using multivariable analyses adjusted for sex, age, dyslipidemia, diabetes, hypertension, obesity, estimated glomerular filtration rate, and alcohol consumption.

**Results:**

During the follow-up (median six years), we recorded 341 (0.3%) new atrial fibrillation cases. Univariable analysis showed a significant association between QTc prolongation and variability with new-onset atrial fibrillation (odds ratio [OR] per 10 ms, 1.08 and 1.15; 95% confidence interval [CI], 1.03–1.13 and 1.07–1.23, respectively; P < 0.001). Multivariable analysis suggested increased risk of new-onset atrial fibrillation with QTc prolongation and QT variability (OR per 10 ms, 1.09 and 1.16; 95% CI, 1.04–1.14 and 1.09–1.24, respectively; P < 0.001).

**Conclusion:**

In the general Japanese population, QTc prolongation and large QT variability are risk factors for new-onset atrial fibrillation. QT prolongation and large QT variability during sinus rhythm may be good markers for predicting not only ventricular but also atrial arrhythmias such as atrial fibrillation.

## Introduction

The overall population aged ≥65 years has risen from 5.1% in 1950 to 9.3% in 2020 and is projected to increase further to 17.8% in 2060. This indicates that the population will age rapidly over the next 40 years [1]. Atrial fibrillation (AF) is associated with adverse cardiovascular outcomes, such as heart failure, myocardial infarction, stroke, and mortality [2]. Thus, it is vital to examine the AF prevalence and its associated risk factors, such as advanced age, diabetes, obesity, hyperuricemia, sleep apnea syndrome, hypertension, smoking, and habitual alcohol drinking [3,4]. Previously, we reported a significant association between abdominal obesity and new-onset AF incidence in males [5]. Early detection of modifiable risk factors for AF, such as abdominal obesity, can improve patient quality of life and prognosis.

Age and obesity are non-electrocardiographic risk factors for AF. Other electrocardiographic markers that predict AF include the PR interval [6] and premature atrial contractions [7]. However, we focused on the QT interval (QTc). QTc is suggestive of ventricular repolarization and is a common predictor of ventricular arrhythmias. Prolonged QTc and AF development are likely linked [8], indicating an association between the QTc and ventricular and atrial arrhythmias. However, reports on QTc variability and AF development are limited. Therefore, we explored whether any discernible association exists in the Japanese population between QTc prolongation, QT variability, and new-onset AF.

## Materials and methods

### Study design

This retrospective study analyzed data from all participants who underwent annual health screenings at least twice to evaluate QTc variability between April 2005 and October 2018 at the Kagoshima Kouseiren Medical Healthcare Center (Kagoshima, Japan). Most participants’ annual health examinations were performed according to the recommendations of the Japanese health welfare policy. Data were collected using self-administered questionnaires on medical history, including diabetes mellitus, dyslipidemia, hypertension, medication history, and alcohol consumption. Each participant’s blood pressure was measured after sitting quietly for 5 min. AF was diagnosed using only a 12-lead surface electrocardiogram (ECG) during each annual health checkup. After an overnight fast, blood samples were collected.

Cardiovascular risk factors encompassed the following: diabetes: fasting blood glucose ≥126 mg/dL, or active treatment with oral hypoglycemic agents or insulin; hypertension: systolic blood pressure ≥140 mmHg and/or diastolic blood pressure ≥90 mmHg or ongoing use of antihypertensive medications; dyslipidemia: serum triglycerides ≥150 mg/dL, serum low-density lipoprotein cholesterol ≥140 mg/dL, serum high-density lipoprotein cholesterol <40 mg/dL, or the use of lipid-lowering agents [9].

Alcohol consumption for ≥10 days per month was considered habitual drinking. Obesity was described as a body mass index (BMI) of >25 kg/m2. The estimated glomerular filtration rate (eGFR) was calculated using the following formula: eGFR (mL/min/1.73 m2) = 194 × serum creatinine−1.094 × age−0.287 × 0.739 (female) [10]. An eGFR < 60 indicated chronic kidney disease. The Bazett formula (QTc = QT/√RR), which uses the mean QT and RR intervals, was applied to calculate the QTc interval using automatic analysis of electrocardiograms. QTc variability during the follow-up period was defined as the difference between the maximum and minimum QTc values, as previously described [11].

### Inclusion and exclusion criteria

**Fig 1:**
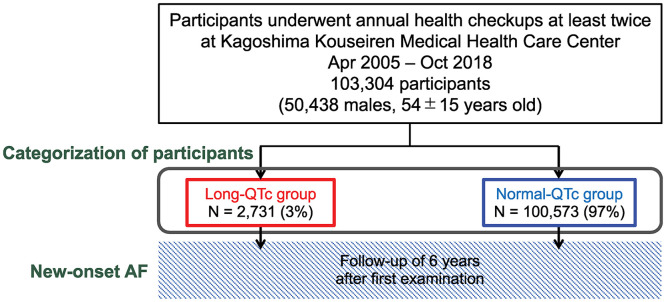
Flowchart showing the study participant enrollment process.

Fig 1 presents a flowchart of the enrollment process. Patients with AF at the time of initial and second screening or diagnosis before the first health screening were removed from the study. Data from the initial checkup at baseline were selected, and individuals with data for approximately 6 years (range, 3–8 years) were included. Patients with premature ventricular contractions, supraventricular premature contractions, or right or left bundle branch block on electrocardiogram were excluded.

### Ethics approval

The Kagoshima University Graduate School of Medical and Dental Sciences Institutional Ethics Committee approved this retrospective study (approval no. 170130(520), dated December 13, 2021). The informed consent requirement was waived because anonymized data were analyzed. The study protocol was in accordance with the principles of the Declaration of Helsinki. The authors had no access to information that could identify individual participants during or after data collection. The data was accessed on February 13, 2023.

### Categorization of participants

The participants were segregated into two groups based on sex and QTc time. Normal QTc values for males and females are ≤ 440 ms and ≤460 ms, respectively [12]. Therefore, males with QTc > 440 ms and females with QTc > 460 ms formed the Long-QTc group, whereas the remaining participants formed the Normal-QTc group (Fig 1). The participants were also divided into two groups based on the median QTc variability, which was 17.1 ms for males and females. Those with QTc variability >17.1 ms were classified into the Long-QTc group, whereas the rest were placed in the Normal-QTc group (S1 Table).

### Data analysis

The relationships between QT prolongation and variability and new-onset AF were determined using logistic regression analysis.

### Multiple regression analysis

Multivariable analyses were adjusted for clinical variables (sex, age, diabetes, dyslipidemia, hypertension, eGFR, obesity, and habitual alcohol consumption) for which significant differences were observed between the two groups in Table 1.

**Table 1:** Participants’ Baseline Characteristics Stratified by QTc Time.

	All	Long-QTc	Normal-QTc	P-value
	(n = 103304)	(n = 2731)	(n = 100573)	(Long vs. Normal)
Age (years)	53.7 ± 14.7	60.3 ± 13.2	53.5 ± 14.7	<0.001
Sex (Male)	50438 (48.8)	1769 (64.8)	48669 (48.4)	<0.001
BMI (kg/m2)	23.2 ± 3.5	23.9 ± 3.9	23.2 ± 3.4	<0.001
Habitual drinking	23722 (23.0)	562 (20.6)	23160 (23.0)	<0.01
Hypertension	34394 (33.3)	1596 (58.4)	32798 (32.6)	<0.001
SBP (mmHg)	124.8 ± 19.0	136.7 ± 20.2	124.4 ± 18.9	<0.001
DBP (mmHg)	75.9 ± 11.4	81.0 ± 12.4	75.8 ± 11.3	<0.001
Diabetes	7023 (6.8)	355 (13.0)	6668 (6.6)	<0.001
Dyslipidemia	47511 (46.2)	1440 (52.9)	46071 (46.0)	<0.001
Total cholesterol (mg/dL)	206.2 ± 35.3	203.0 ± 35.9	206.3 ± 35.3	<0.001
Triglyceride (mg/dL)	88 (63, 128)	102 (69, 152)	88 (62, 127)	<0.001
LDL-C (mg/dL)	124.6 ± 32.0	120.5 ± 33.3	124.7 ± 31.9	<0.001
HDL-C (mg/dL)	60.8 ± 14.8	58.3 ± 15.5	60.8 ± 14.8	<0.001
eGFR (mL/min/1.73m2)	80.5 ± 16.3	77.5 ± 17.3	80.6 ± 16.3	<0.001
Heart rate (beats/min)	65.4 ± 10.1	72.8 ± 13.3	65.2 ± 9.9	<0.001
QTc (ms)	406.1 ± 22.4	458.5 ± 17.4	404.7 ± 20.7	<0.001

Values are presented as mean ± standard deviation or n (%).

BMI, body mass index; SBP, systolic blood pressure; DBP, diastolic blood pressure; LDL-C, low-density lipoprotein cholesterol; HDL-C, high-density lipoprotein cholesterol; eGFR, estimated glomerular filtration rate.

### Statistical analyses

Continuous variables are stated as mean ± standard deviation, whereas triglyceride values were expressed as median (first quartile – third quartile). Categorical variables are presented as absolute numbers and percentages. Categorical variables were compared using the chi-square test. Group comparisons of continuous variables were performed by a two-sample t-test or the Wilcoxon rank-sum test. Statistical significance was set at P < 0.05. Statistical analysis was carried out using JMP Pro v16 (SAS Institute, Inc., Cary, NC, USA).

## Results

### Participant baseline characteristics

Between April 2005 and October 2018, 115,462 individuals participated in at least two annual health checkups. After applying the exclusion criteria, 12,158 individuals were excluded (10,163 lacking data and 1,995 with underlying cardiac disease), resulting in the analysis of data from 103,304 participants. The participant enrollment flowchart is shown in Fig 1. Table 1 presents the baseline characteristics of participants arranged based on their QTc interval. Significant differences were observed in the Long-QTc group, including higher age, BMI, and heart rate, as well as higher rates of male sex, diabetes, dyslipidemia, hypertension, and chronic kidney disease. The baseline characteristics of participants categorized based on their QTc variability are presented in S1 Table.

### Distribution of QTc time and QTc variability

**Fig 2:**
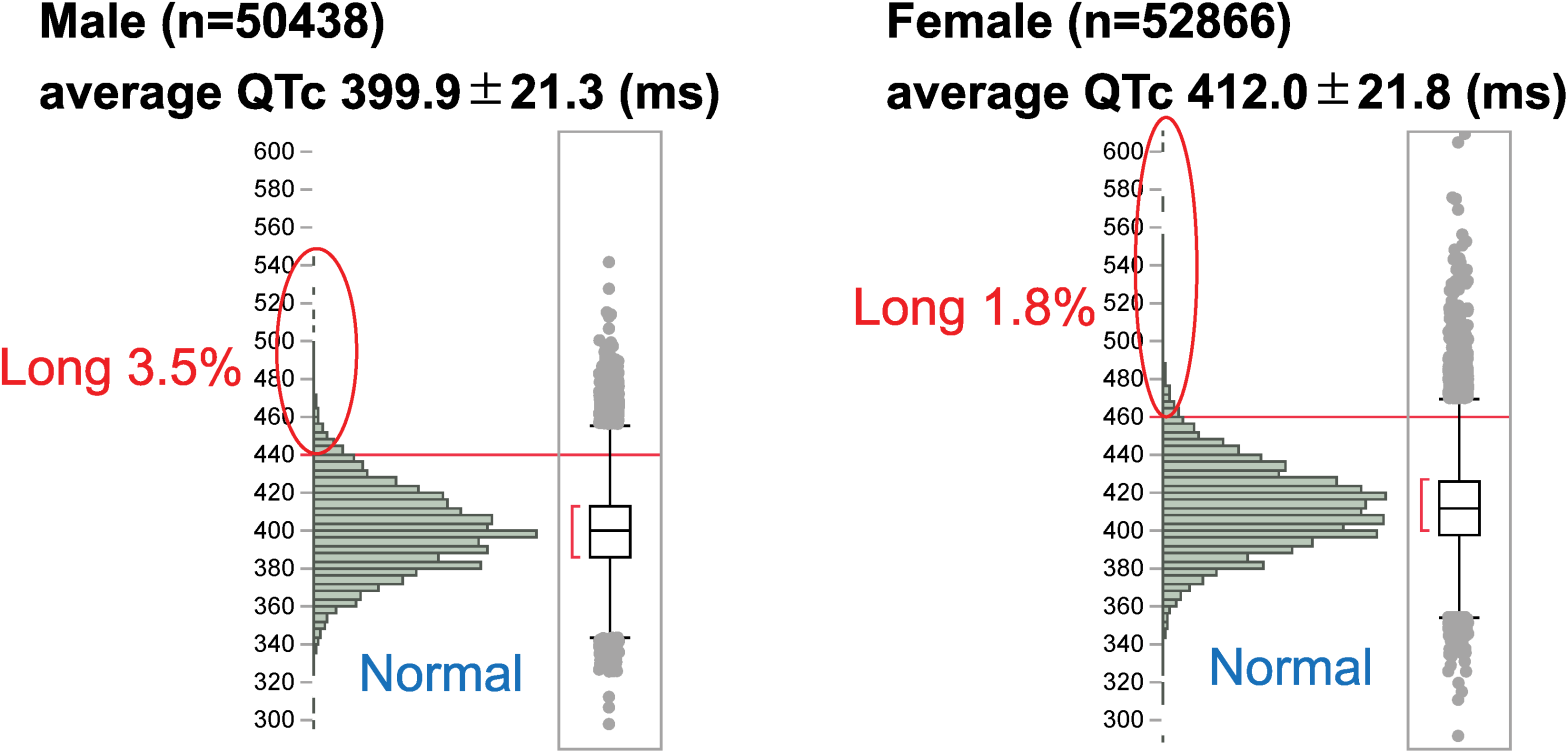
Distribution of QTc time.

QTc distribution is shown in Fig 2. Only 3.5% of males and 1.8% of females were in the Long-QTc group. The average QTc was 399.9 ± 21.3 ms for males and 412.0 ± 21.8 ms for females.

The distribution of QTc variability is shown in S1 Fig. The average QTc variability was 19.1 ± 12.5 ms for males and 19.4 ± 13.4 ms for females.

### Sex differences in the incidence of AF

During the follow-up (median 6 years), 341 (0.3%) new individuals were diagnosed with AF. There were sex differences in the incidence of AF; however, the incidence of AF was significantly higher in the Long-QTc group for males and females (Table 2).

**Table 2:** Sex Differences in the Incidence of Atrial Fibrillation.

	Males (n = 253)	Females (n = 88)
Long-QTc group	1.07%	0.52%
	(19/1769)	(5/962)
Normal-QTc group	0.48%	0.15%
	(234/48669)	(83/51904)
(Long vs. Normal)	P < 0.001	P = 0.02

In addition, some individuals exhibited extremely long or short QTc values (>600 ms or <300 ms). Therefore, we excluded 69 individuals (47 males) with QTc less than 300 ms or greater than 600 ms and performed a reanalysis. The results also showed a significantly higher incidence of AF in the Long-QTc group for both sexes (males: Long vs. Normal, 1.07% vs. 0.48%, P < 0.001; females: 0.52% vs. 0.16%, P = 0.02).

### Univariable and multivariable logistic regression analyses of the predictors of new-onset AF

Table 3 displays the univariable and multivariable logistic regression analysis results, which identified the predictors of new-onset AF. Univariable analysis showed a significant increase in new-onset AF for QTc prolongation (odds ratio [OR] per 10 ms, 1.08; 95% confidence interval [CI], 1.03–1.13; P < 0.001) and QTc variability (OR per 10 ms, 1.15; 95% CI, 1.07–1.23; P < 0.001). Following adjusting for clinical variables in multivariable analysis, QTc prolongation and variability remained significantly associated with new-onset AF (OR per 10 ms, 1.09; 95% CI, 1.04–1.14; P < 0.001) (OR per 10 ms, 1.16; 95% CI, 1.09–1.24; P < 0.001). Moreover, as per multivariable analysis, the long-QTc group was associated significantly with new-onset AF (OR 1.56; 95% CI, 1.02–2.39; P < 0.05). Multivariable analysis revealed that QT prolongation and variability, age, sex, obesity, and habitual alcohol consumption were independent predictors of new-onset AF.

**Table 3:** Predictors of New-Onset Atrial Fibrillation.

	Univariable analysis		Multivariable analysis	
	OR (95% CI)	P-value	OR (95% CI)	P-value
Long-QTc group	2.80(1.85-4.25)	<0.001	1.56(1.02-2.39)	0.042
QTc variability (10 ms)	1.15(1.07-1.23)	<0.001	1.16(1.08-1.24)	<0.001
Age (years)	1.06(1.05-1.07)	<0.001	1.06(1.05-1.08)	<0.001
Sex (Male)	3.02(2.37-3.85)	<0.001	2.64(2.02-3.45)	<0.001
Obesity	1.52(1.22-1.90)	<0.001	1.46(1.16-1.84)	0.001
Hypertension	2.50(2.02-3.10)	<0.001	1.20(0.95-1.52)	0.117
Dyslipidemia	1.07(0.86-1.32)	0.559	0.91(0.73-1.13)	0.396
Diabetes	1.93(1.40-2.67)	<0.001	1.15(0.83-1.59)	0.414
Chronic kidney disease	1.58(1.14-2.18)	0.006	0.77(0.55-1.08)	0.134
Habitual drinking	2.46(1.98-3.05)	<0.001	1.83(1.44-2.32)	<0.001

## Discussion

This study reported that prolonged QT and excessive variability in QT are associated with new-onset AF based on health checkup data from the Japanese population. Reportedly, QT prolongation is associated with new-onset AF [8,13,14]; however, the association between QT variability and new-onset AF has rarely been reported [11]. Magnano et al. reported an association between QT variability on Holter ECGs and new-onset AF [11]. They analyzed 21 Holter ECGs with 2500 beats to examine the onset of AF and its characteristics using QT measurements. However, this is the first study to report on the association between QT variability and AF using health checkup data.

In the present study, the frequency of new-onset AF was significantly higher in the Long-QTc group than in the normal-QTc group. However, as noted in the patient background, the Long-QTc group had significantly higher rates of older age, obesity, diabetes, dyslipidemia, hypertension, and chronic kidney disease than the Normal-QTc group. Therefore, multivariable analysis revealed that QT prolongation and variability were independent predictors of new-onset AF; however, the involvement of confounding factors cannot be ruled out. Since our study was an analysis of health checkup results, left atrial diameter and left ventricular hypertrophy were not evaluated by ECG. However, the Long-QTc group may have a higher frequency of new-onset AF as a result of structural remodeling of the atrium caused by external stress, such as hypertension and diabetes mellitus [15].

In addition to QT prolongation and variability, age, sex, obesity, and habitual alcohol consumption could independently predict new-onset AF following multivariable analysis (Table 3). We have previously focused on abdominal circumference in obesity and analyzed the association between abdominal circumference and new-onset AF using health checkup data [5] similar to the present study. Abdominal circumference ≥85 cm in males and ≥90 cm in females was considered abdominal obesity. A sex difference was observed regarding the association between abdominal obesity and new-onset AF in males. In contrast, the frequency of new-onset AF was significantly higher in males and females in the Long-QTc group, and no sex difference was observed in the incidence of AF in the Long-QTc group, with a male AF incidence rate of 1.07% and a female AF incidence rate of 0.52%. Therefore, QT is a risk factor for AF in males and females.

Ishikawa et al. reported an association between QTc and future stroke, finding a 2.7-fold increased risk of stroke in patients with QT prolongation without left ventricular hypertrophy on ECG when compared with those with neither QT prolongation nor left ventricular hypertrophy on ECG [16]. The study did not evaluate the presence or absence of AF during follow-up; however, our results suggest that the incidence of AF during follow-up was more frequent in the group with QT prolongation and may have increased the risk of stroke.

The biological mechanisms underlying the relationship between QTc prolongation, QTc variability, and new-onset AF have not been clearly elucidated. This may be explained by an “atrial torsades de pointes” mechanism, similar to how ventricular arrhythmias are more likely to occur in long-QT syndrome [14]. Supporting this hypothesis, a mouse model of long QT syndrome type 3 showed that long atrial action potential duration and the triggering of early posterior depolarization are substrates that promote AF [17]. The autonomic nervous system plays a central role in developing AF and is thought to have a significant influence on QT variability. Previously, increased vagal tone was thought to be involved in the development of AF; however, Zimmerman et al. demonstrated that autonomic fluctuations favor either vagal or sympathetic tone before the onset of AF [18]. Thus, statistically different changes in autonomic nervous system activity have been demonstrated depending on the low frequency/high frequency (LF/HF) ratio before arrhythmic episodes [19]. This study did not assess the LF/HF ratio on Holter ECGs; however, it showed that QT variability may be related to autonomic fluctuations. We also compared the two groups in terms of patient background, even in terms of QT variability (S1 Table). The group with greater QT variability had a significantly higher heart rate, suggesting a link between QT variability and autonomic nervous system fluctuations.

Nielsen et al. reported that QT shortening is also a risk factor for AF, in addition to QT prolongation. This study focused on QT prolongation and QT variability; however, further research is needed to explore the relationship between QT shortening and AF [20].

The study had some limitations. First, selection bias was likely to have occurred in this retrospective observational study. Second, the detection rate of AF might have been underestimated because it was symptomatic, and monitoring was not continuous. Third, the lower incidence of new-onset AF, especially among females, might have limited the power of the association between new-onset AF and QT prolongation and variability. Fourth, the annual physical examination data make time analysis difficult. Finally, although the health checkup data revealed the presence of oral treatment for diabetes, dyslipidemia, and hypertension, the details of the medications were unknown. For example, if the patient was taking an oral diuretic, it could have caused low potassium, resulting in QT prolongation and AF. In addition to the lack of information on medications, there was no information on the electrolytes that cause QT prolongation, such as potassium. Further investigations with longer follow-up periods are required to validate the present findings.

In conclusion, as reflected in the health checkup data from the general Japanese population, QTc prolongation and variability can indicate a heightened risk of new-onset AF. QT prolongation and large QT variability during sinus rhythm may be good markers for predicting not only ventricular but also atrial arrhythmias, such as AF.

## Supporting information

S1 Table Participants’ Baseline Characteristics Stratified by QTc Variability. (DOCX)

S1 Fig Distribution of QTc variability among participants. (TIF)
