# Supplementary material for: QT prolongation and excessive variability predicts new-onset atrial fibrillation in the health screening data of Japanese adults
Source: PLoS One. 2025 Oct 22;20(10):e0333169. doi: 10.1371/journal.pone.0333169 (PMC12543107; doi:10.1371/journal.pone.0333169)
Supplement: S1 Table — (DOCX) [file pone.0333169.s001.docx]

**S1 Table. Participants’ Baseline Characteristics Stratified by QTc Variability.**

|  | All | Large-QTc | Normal-QTc | P-value |
| --- | --- | --- | --- | --- |
|  | (n=103304) | (n=51654) | (n=51650) | (Large vs. Normal) |
| Age (years) | 53.7±14.7 | 52.8±14.8 | 54.7±14.5 | <0.001 |
| Sex (Male) | 50438 (48.8) | 25298 (49.0) | 25140 (48.7) | 0.331 |
| BMI (kg/m^2^) | 23.2±3.5 | 23.3±3.5 | 23.2±3.4 | <0.001 |
| Habitual drinking | 23722 (23.0) | 12903 (25.0) | 10819 (21.0) | <0.001 |
| Hypertension | 34394 (33.3) | 16879 (32.7) | 17515 (33.9) | <0.001 |
| SBP (mmHg) | 124.8±19.0 | 124.7±18.9 | 124.8±19.0 | 0.269 |
| DBP (mmHg) | 75.9±11.4 | 76.2±11.4 | 75.7±11.3 | <0.001 |
| Diabetes | 7023 (6.8) | 3459 (6.7) | 3564 (6.9) | 0.193 |
| Dyslipidemia | 47511 (46.2) | 23693 (46.1) | 23818 (46.4) | 0.266 |
| Total cholesterol (mg/dL) | 206.2±35.3 | 206.7±35.4 | 205.8±35.1 | <0.001 |
| Triglyceride (mg/dL) | 88 (63, 128) | 87 (62, 127) | 88 (63, 128) | 0.070 |
| LDL-C (mg/dL) | 124.6±32.0 | 124.9±32.1 | 124.3±31.9 | 0.004 |
| HDL-C (mg/dL) | 60.8±14.8 | 61.0±14.8 | 60.5±14.7 | <0.001 |
| eGFR (mL/min/1.73m^2^) | 80.5±16.3 | 81.3±16.5 | 79.7±16.0 | <0.001 |
| Heart rate (beats/min) | 65.4±10.1 | 65.9±10.3 | 65.0±9.8 | <0.001 |
| QTc (ms) | 406.1±22.4 | 404.2±24.4 | 408.1±20.0 | <0.001 |

BMI, body mass index; SBP, systolic blood pressure; DBP, diastolic blood pressure; LDL-C, low-density lipoprotein cholesterol; HDL-C, high-density lipoprotein cholesterol; eGFR, estimated glomerular filtration rate.
