# Supplementary figures and images for: QT prolongation and excessive variability predicts new-onset atrial fibrillation in the health screening data of Japanese adults
Source: PLoS One. 2025 Oct 22;20(10):e0333169. doi: 10.1371/journal.pone.0333169 (PMC12543107; doi:10.1371/journal.pone.0333169)

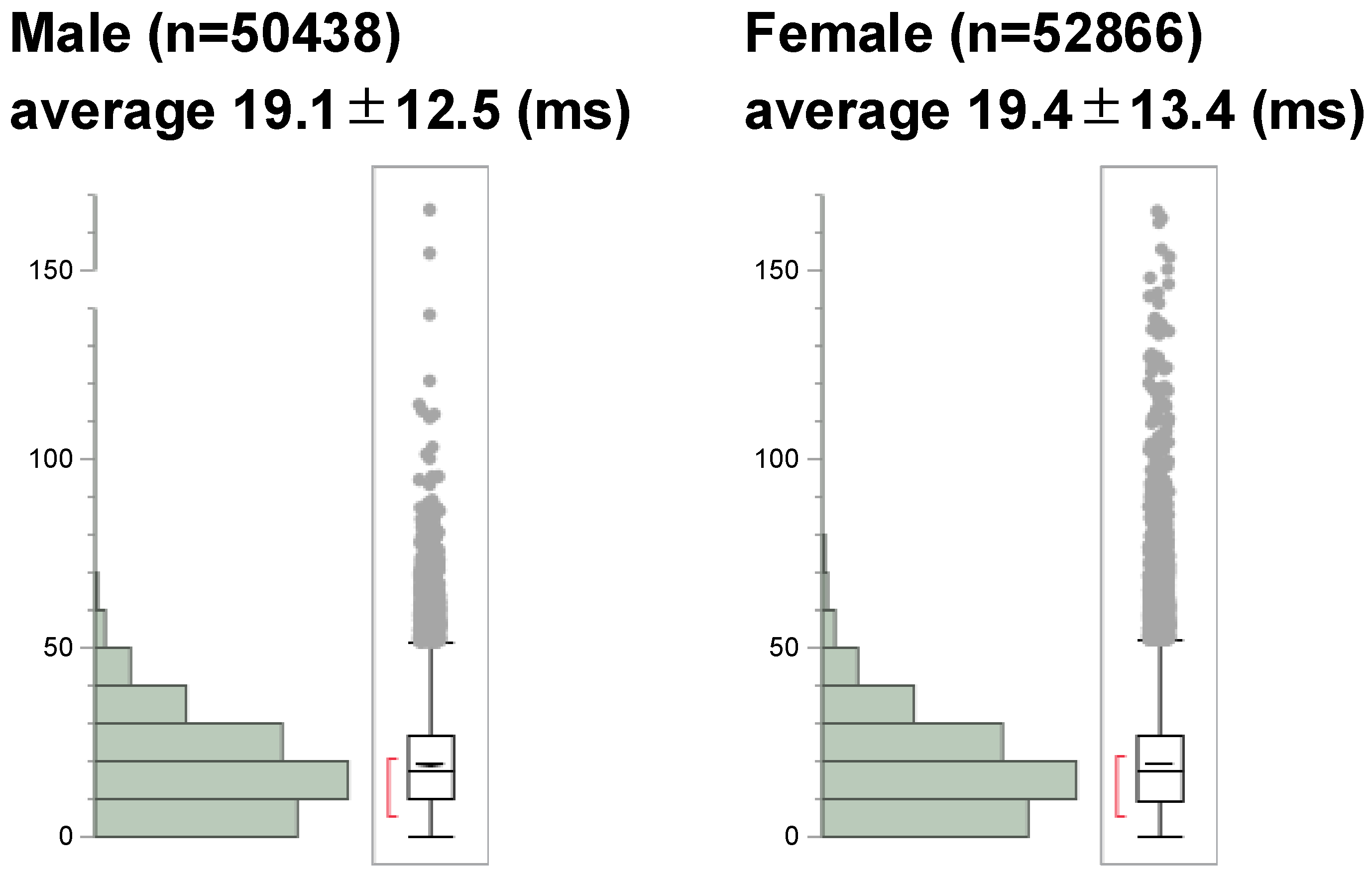

Supplement: S1 Fig — (TIF) [file pone.0333169.s002.tif]
